# The water footprint of lithium extraction technologies: Insights from environmental impact reports in Argentina's salt flats

**DOI:** 10.1016/j.heliyon.2025.e42523

**Published:** 2025-02-07

**Authors:** Walter Fernando Díaz Paz, Lucas Seghezzo, Ariela Griselda Salas Barboza, Melisa Escosteguy, Paula Valentina Arias-Alvarado, Eduardo Kruse, Marc Hufty, Martín Alejandro Iribarnegaray

**Affiliations:** a Consejo Nacional de Investigaciones Científicas y Técnicas (CONICET), Universidad Nacional de Salta (UNSa), Avenida Bolivia 5140, A4408FVY, Salta, Argentina; b UNSa, Avenida Bolivia 5140, A4408FVY, Salta, Argentina; c Centro de Investigaciones y Transferencia del Noroeste de la Provincia de Buenos Aires (CIT-NOBA), CONICET, Universidad Nacional de La Plata (UNLP), Casco Urbano, B1900, La Plata, Provincia de Buenos Aires, Argentina; d Centre for International Environmental Studies, Geneva Graduate Institute, 2 Ch. Eugene-Rigot, CH1211, Geneva, Switzerland

**Keywords:** Brine consumption, Freshwater consumption, Lithium extraction, Population equivalent, Water footprint

## Abstract

This study estimates water consumption in two lithium mines (Olaroz and Fénix) that use different extraction technologies in Argentina's salt flats. Based on Environmental Impact Reports (EIRs), we assess the water footprint (WF) and brine consumption (BC) in both mines. To the best of our knowledge, this study is the first to estimate WF and BC for lithium extraction and provides data to assess water consumption and better understand its implications for local ecosystems and communities. We also contextualize freshwater consumption in lithium extraction projects by estimating the blue water intensity (WI_blue_) and the population equivalent (PE), namely the number of local inhabitants that would consume an equivalent volume of water. Total WF was 51.0 and 135.5 m^3^/ton of lithium carbonate (Li_2_CO_3_) for Olaroz and Fénix, respectively. Per unit of product, WF was 2.7 times higher in Fénix but BC was higher in Olaroz. WI_blue_ indicates that, while Fénix had a higher WF_blue_, its impact on local blue water availability is moderate due to greater local water availability. WF_blue_ in Olaroz and Fénix was equivalent to the water consumption of 32,238 and 141,047 inhabitants of their nearest towns (Susques and Antofagasta de la Sierra, respectively, both with a current population of less than 2,100 inhabitants). Our findings underscore that the water consumption of lithium mining can have important impacts that vary significantly with geographic context. EIRs provide a useful basis for estimating WF and BC, though certain limitations and challenges persist, particularly regarding incomplete or insufficiently detailed data.

## Highlights

• Water footprint and brine consumption were estimated for two lithium mines.
• The Fénix mine had a 2.7 times higher water footprint per ton of lithium carbonate.
• Brine consumption per unit of product was higher in Olaroz than in Fénix.
• The water footprint was equivalent to 32,238 and 141,047 local inhabitants' use.
• Environmental impact reports are useful but have data limitations in water use.

## 1 Introduction

“The world will soon demand lithium batteries with the least possible water footprint”, said Rodolfo García Maurizzio, a locally renowned hydrogeologist, addressing hundreds of members of lithium mining companies, government officials, and scholars during an annual conference held in Salta, Argentina, at the end of 2023. He went on to coin the term *hydro-fantasies*, which he defined as “statements made by professionals in the lithium mining industry regarding something they are not really familiar with: the functioning of the water system”.^1^

In fact, the hydrological systems of the basins and catchments where lithium extraction projects are located are not well understood [1,2]. Yet the number of lithium extraction projects in northern Argentina's salt flats continues to grow, raising concerns about the potential environmental and social impacts related to water consumption in an area that is essentially a desert [[3], [4], [5]]. Mining companies and government officials argue that lithium extraction requires less water than other activities like agriculture or other types of mining, such as for gold and copper [[6], [7], [8]]. This debate highlights the need for a more comprehensive approach that better quantifies water consumption and considers other competing water uses [[9], [10], [11]].

However, establishing a standardized methodology to measure water consumption faces many challenges [12,13]. Marconi et al. (2022) [14] highlights the importance of examining water consumption in lithium extraction at a basin scale, considering cumulative and synergistic impacts. Marchegiani et al. (2019) [15], Escosteguy et al. (2022) [16], Pragier et al. (2022) [17], and Escosteguy et al. (2023) [18] reported the existence of local concerns related to water consumption in lithium extraction, in addition to controversies about the unequal distribution of economic benefits and scarce participation in consultation processes. Blair et al. (2024) [2] pointed out that local concerns about water consumption are not primarily related to the volume of water consumed but rather to the uncertainty surrounding the present and future impacts of this water consumption. Despite recurring studies of water resources and lithium extraction, only a few studies examined the implications of water consumption in the context of multiple water and territory uses, water scarcity, and the upcoming expansion of lithium extraction projects [[19], [20], [21]].

Based on previous works, water consumption of lithium extraction ranges from approximately 5 to 50 m^3^ of freshwater per ton of lithium carbonate (Li_2_CO_3_), with an additional 500 m^3^ during the evaporative brine concentration phase [22,23]. Deniau et al. (2021) [24] suggest a total water consumption of around 2,000 m^3^ per ton of Li_2_CO_3_. Corporate sustainability reports, however, provide a “water intensity index” ranging from 48.0 to 71.3 m^3^ per ton of Li_2_CO_3_ [6,7]. Vera et al. (2023) [25] propose that water consumption in lithium extraction strongly depends on technical and environmental factors as well as production technology. Technologies currently grouped as Direct Lithium Extraction (DLE) may require larger volumes of freshwater compared to existing evaporative practices, potentially limiting its suitability and acceptability in the predominantly arid regions where lithium extraction takes place [26]. In any case, debating the high or low value of water consumption in lithium extraction or any industry cannot be analyzed solely based on physical magnitudes and a contextualized environmental and social analysis should be provided for each case study [9,13,27].

The water footprint (WF) is a volumetric measure of the freshwater consumption across the production processes of goods and services that are consumed by a given population (individual, regional, national, or global) for a certain period [28]. This methodology, extensively validated across diverse industrial sectors globally, provides a robust analytical framework to gauge water consumption in a more comprehensive way [11,29,30]. Beyond quantifying water embodied in the production process, the WF enables a contextualized analysis, putting water consumption in perspective with respect to local water supply and competing uses [28,31]. Based on a life cycle assessment (LCA) approach, the international WF standard (ISO 14046) defines the WF as a metric that quantifies potential environmental impacts related to water [32]. The ISO methodology introduces environmental impact equivalents that reflect the pressure exerted by water consumption on water resources [33]. While the ISO methodology aligns in certain aspects with that of Hoekstra et al. (2011) [28], significant conceptual and empirical differences still exist [32,35]. Based on the ISO WF concept, the International Lithium Association (ILiA) - the main industry association of lithium - is developing a specific methodological guide to quantify “the Product Water Scarcity Footprint for Lithium Products” (PWSF) [34]. In this study, we decided to use the methodology of Hoekstra et al. (2011) [28], which defines WF as a volumetric measure of water appropriation, as it is better suited for assessing WF at the basin scale. This approach considers water appropriation as the volume consumed in production processes that is no longer available for other local uses, which is particularly relevant in the specific context of our study.

The WF includes three different components: (1) the blue water footprint (WF_blue_); (2) the green water footprint (WF_green_); and (3) the gray water footprint (WF_gray_). The WF_blue_ is the volume of freshwater consumed, i.e. water extracted from surface or groundwater basins that does not return to the basin in the form of return flow. The WF_green_ is the volume of freshwater consumed via evapotranspiration from the land surface, mostly for growing crops or production forests. The WF_gray_ is the volume of freshwater required to assimilate the load of pollutants based on natural background concentrations and existing ambient water quality standards [28]. As a whole, the WF measures the volume of freshwater that is no longer available for further use or for the ecosystem within the basin from which it has been withdrawn [36].

The WF has been estimated in different sectors and activities, including agriculture and livestock [[37], [38], [39]], the textile industry [40], the beverage industry [41,42], and copper mining [12,43,44], among many others. This wide application underscores the versatility, practicality, and sensitivity of this indicator. In each case, in-depth understanding of the production process, coupled with a clear goal and scope setting, is essential for accurate measurement [28,45]. The WF estimation depends heavily on the availability of baseline data, which is often either unavailable or not freely accessible [46,47]. Therefore, it is essential to develop open-access data systems to advance WF estimation, thereby generating reliable and transparent information to guide decision-making regarding water use [48,49]. We based our study on data from the Environmental Impact Reports (EIRs) that mining companies submit to provincial authorities as part of the environmental impact assessment process. In Argentina, the EIR is the document on which the provincial mining authority bases its decision on a mining project [50].

In this work, we estimated the WF of the production of battery-grade lithium carbonate (Li_2_CO_3_) for two lithium extraction projects (Olaroz and Fénix) with different types of extraction technologies from salt flats. We also calculated brine consumption (BC) and discussed the relation with WF. To the best of our knowledge, this work represents the first estimation of the WF for lithium extraction using EIRs data. We hypothesize that EIRs can provide accurate empirical data for estimating the lithium extraction WF, thereby contributing to a better understanding of its water consumption and socio-environmental impacts on local ecosystems and communities.

## 2 Methodology and data sources

### 2.1 Brief description of the Argentine Puna

**Fig. 1:**
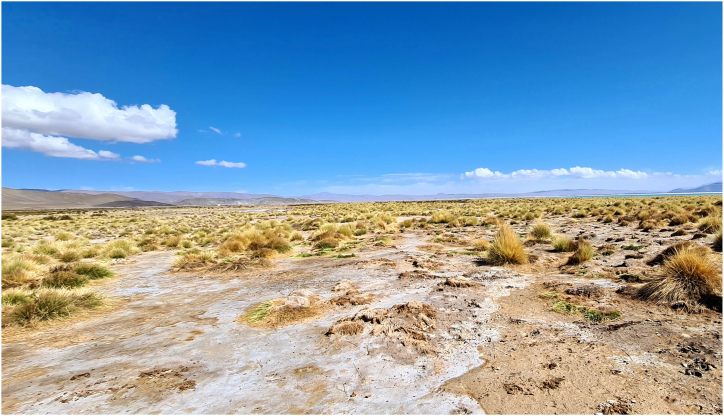
Typical Puna region landscape around the Hombre Muerto salt flat in the province of Catamarca (Argentina).

Lithium extraction projects in Argentina are situated in the northwestern region of the country, in a region known as the Puna. The Argentine Puna is a high-altitude plateau that covers around 125,000 km^2^ [51,52] (Fig. 1). The presence of mountain systems with north-south distribution and elevations above 5,000 m above sea level define an arid regional climate [53]. Due to orographic effects, rainfall gradually decreases from east to west and from north to south in the region, ranging from approximately 350 mm per year in the northeast to less than 50 mm per year in the southwest [19]. Temperatures can be extreme, going up to 30 °C or more during the day in summer and below −20 °C on winter nights [54]. The Puna region also has one of the highest solar radiations in the world [55]. Low rainfall, daily thermal amplitude, high intensity of solar radiation, permanent winds, and low relative humidity generate evapotranspiration rates between 2,000 and 2,500 mm/year, which results in a permanent negative hydrological balance [56]. Throughout the Puna, there are several lagoons and extensive salt flats as a result of long-term dilution and accumulation processes of chemical elements present in Ordovician volcanic rock and deposits of tertiary evaporites [57,58]. Rain and surface water dissolve these elements, which later precipitate by evaporation and accumulate in low-lying areas, leaving large deposits of different salts [59]. Among these elements, lithium and boron are the most economically relevant, and they are generally found associated with potassium and magnesium [60].

### 2.2 Case studies

**Fig. 2:**
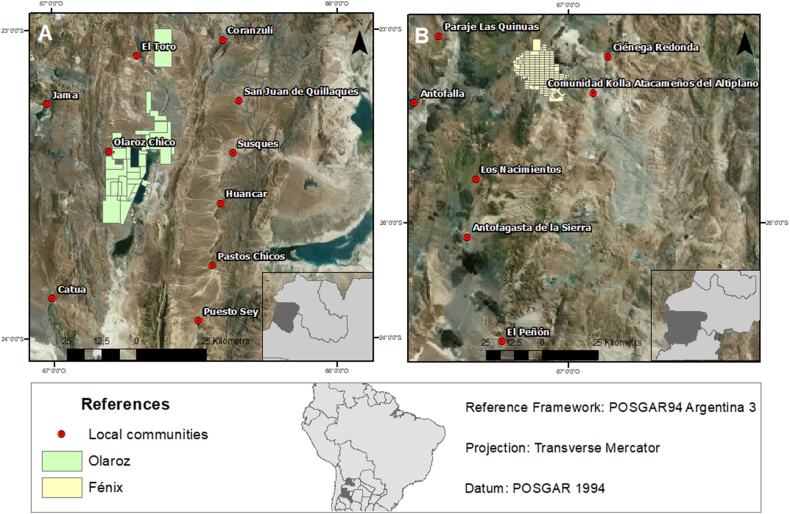
Case studies: (A) Olaroz project, province of Jujuy; and (B) Fénix project, province of Catamarca (Argentina).

We assessed two lithium projects in full production phase in Argentina: (1) the Olaroz project, located at the Olaroz salt flat in the Department of Susques, province of Jujuy (Fig. 2a), and (2) the Fénix project, located at the Hombre Muerto salt flat in the Department of Antofagasta de la Sierra, province of Catamarca (Fig. 2b). Olaroz is operated by Sales de Jujuy S.A. (SdJ), a joint venture between the Australian company Allkem (majority shareholder of SdJ, holding 66.5 % of the shares), the Japanesse Toyota Tsusho (holding 25 % of the shares), and the provincial state company Jujuy Energía y Minería Sociedad del Estado (JEMSE) (holding 8.5 % of the shares). The project is completing an expansion phase that is expected to increase the battery-grade Li_2_CO_3_ production capacity up to 25,000 tons per year [7]. In Olaroz, all battery-grade Li_2_CO_3_ production is carried out at the mining facilities built around the Olaroz salt flat.

Fénix is operated by Minera del Altiplano (MdA), the Argentine subsidiary of the American company Livent Corp [6]. The production phase started in 1997, being the first lithium project to operate in Argentina. In Fénix, lithium production from brine yields two products: (1) battery-grade Li_2_CO_3_, and (2) LiCl (lithium chloride). Battery-grade Li_2_CO_3_ is produced at the Fénix mining facilities located in the Hombre Muerto salt flat, while LiCl is obtained in the city of General Güemes, in the province of Salta [61]. In 2022, Fénix launched an expansion phase that is expected to increase its production capacity to about 100,000 tons of battery-grade Li_2_CO_3_ per year by the end of 2030 [61]. In early 2024, Allkem merged with Livent Corp. to form Arcadium Lithium, which now controls both projects. The company became a global leader in the lithium market by controlling not only lithium extraction operations from salt flats but also from rock, as well as the manufacture of lithium-based chemical products [62]. Recently, all the shares of Arcadium Lithium were acquired by Rio Tinto, a British-Australian multinational company that is the world's second largest metals and mining corporation [63].

Both projects rely on brine pumped from salt flats [6,7]. In both cases, freshwater pumped from underground aquifers around the salt flats is required at different stages of the production process [14,64,65]. These aquifers may be free or confined, depending on the permeability of surrounding sediments [66]. The existence of freshwater and brine aquifers in each basin depends on a dynamic equilibrium between water inputs by precipitation and infiltration (recharge) and water lost by evaporation and pumping (discharge) [67]. Brine pumping could alter the natural hydrodynamic regulation in the aquifers, with the associated risk of the salinization of freshwater aquifers and lowering both surface and groundwater levels across long distances [1,68].

Our two case studies overlap with territories inhabited or used by local communities. For centuries, local communities have relied on the natural resources and services provided by their territories, which include water, soil, vegetation, and wildlife, to sustain subsistence and small-scale economic activities [69]. They base their diets on products from camelids (such as llama, vicuña, and guanaco), goats, and sheep, as well as small-scale agriculture [70]. In addition to these activities, there is a significant production of handicrafts, including weaving using wool fibers from vicuñas, llamas, and sheep. All these activities are largely constrained by the availability of freshwater, which is obtained from rivers, lagoons, and *vegas* (wetlands) in areas surrounding the salt flats.

Water use is a controversial issue in the region. For mining companies, water resources are only associated with freshwater (surface and groundwater), which is defined, in a very pragmatic way, as “water for industrial use not fit for human consumption”, while the brine is defined as “a saline aqueous substance” with no other potential use than mining [6,7]. For local communities, water is considered an integral part of the environment around which they have developed cultural features and livelihoods closely related to their identity [71,72]. Local communities depend mainly on freshwater resources for basic needs and livestock consumption, but also on the natural equilibrium between brine and freshwater resources that secure the hydrological balance needed to maintain grasslands and wildlife.

### 2.3 Description of lithium extraction processes

**Fig. 3:**
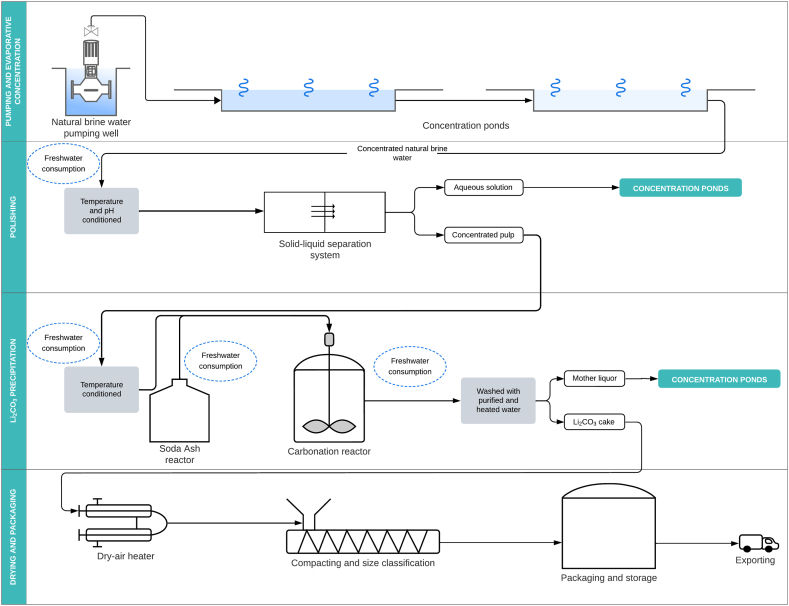
Olaroz project process flowsheet (complete description can be found in the supplementary material). The circles in light blue indicate activities associated with freshwater consumption.

**Fig. 4:**
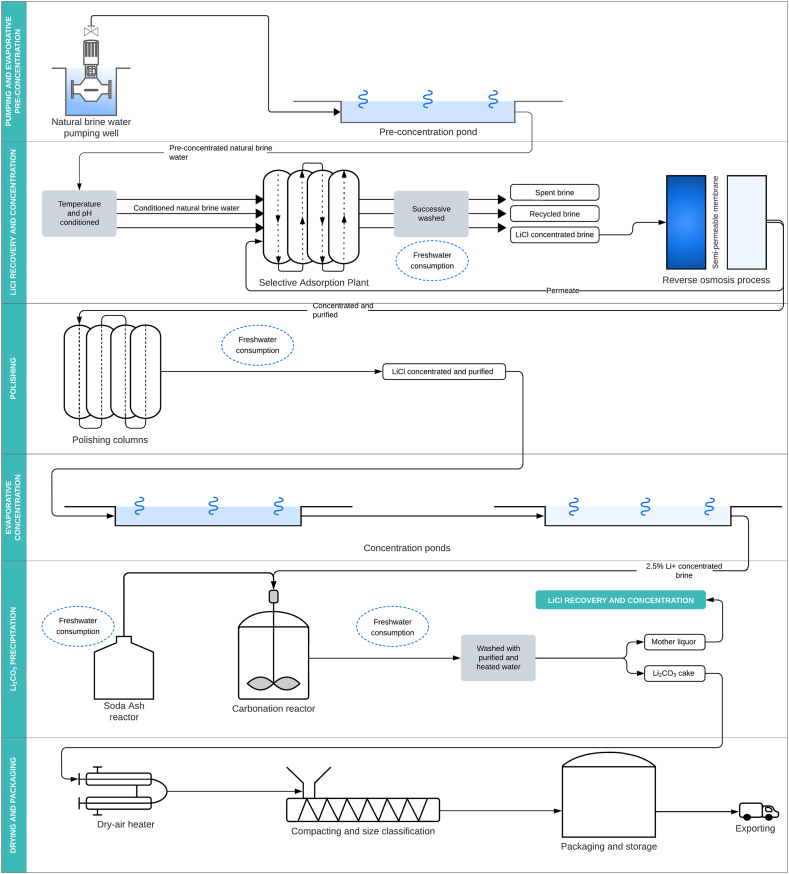
Fénix project process flowsheet (complete description can be found in the supplementary material). The circles in light blue indicate activities associated with freshwater consumption.

The production process at Olaroz is divided into 4 phases (Fig. 3): (1) Pumping and evaporative concentration, (2) Polishing, (3) Li_2_CO_3_ precipitation, and (4) Drying and packaging. The production process at Fénix is divided into 6 phases (Fig. 4): (1) Pumping and evaporative concentration, (2) LiCl recovery and concentration, (3) Polishing, (4) Evaporative concentration, (5) Li_2_CO_3_ precipitation, and (6) Drying and packaging. In both projects, freshwater consumption comes mainly from polishing and Li_2_CO_3_ precipitation. In the polishing phase, the freshwater is used to pre-condition the concentrated brine in terms of temperature and pH to increase the solubility of residual insoluble ions that have not precipitated in the evaporative phase (Ca^2+^, Mg^2+^, Bo^−^, and [AsO_4_]^3-^). In the Li_2_CO_3_ precipitation phase, freshwater is used to prepare a diluted solution of soda ash (Na_2_CO_3_). Soda ash is used to carbonate the concentrated brine and precipitate the Li^+^ ions present, which are recovered as Li_2_CO_3_. In this phase, freshwater is also used to wash and purify the final product to increase purity and Li^+^ ion concentration. In addition to these common phases, freshwater is used at Fénix in the LiCl recovery and concentration phase to wash and remove the Li^+^ ions from the adsorbent columns in the reverse osmosis process.

At Olaroz, the water inflow is freshwater pumped from the Archibarca River underground aquifer. Pumped freshwater is conveyed directly to the production facility through a pipeline system [73]. At Fénix, the water inflow is freshwater pumped from the surface of Trapiche River and its underground aquifer [74]. Surface freshwater is stored in a supply reservoir from which it is conveyed to the production facility through a pipeline system; pumped groundwater is conveyed directly to the production facility [75]. In both case studies, the final product of the production process is battery-grade Li_2_CO_3_ with a lithium purity greater than 99.5 % [6,7]. The water outflow is wastewater generated at the end of the production process, with brackish characteristics due to being a mixture of recycled brine depleted of Li^+^ ions and recycled wash water. According to companies reports, wastewater is conveyed through a pipeline system and discharged into the salt flat surface for natural infiltration. However, previous studies have suggested that infiltration does not occur on the surface of salt flats; instead, water quickly evaporates due to low hydraulic conductivity and compaction caused by the spatial distribution of precipitated salt crystals, which adopt a behavior like that of clays, making the surface impermeable [76].

### 2.4 WF in salt flat lithium extraction

In this study, we estimated the WF for the 2021 production cycle. The WF estimation considered direct freshwater consumption in each production process (described in section 2.3) and the water used for the treatment of domestic wastewater. Indirect water consumption associated with the use of fossil fuel to generate energy, as well as water involved in materials used and the construction of buildings, was excluded. These exclusions were made because they do not draw from local freshwater sources and, therefore, do not impact on the availability of freshwater for other uses.

#### 2.4.1 Data requirements and WF estimation

Information was provided by the Mining and Hydrocarbons Agency of Jujuy and the Provincial Direction of Environmental Mining Management of Catamarca. In both cases, we accessed the EIRs elaborated for the Olaroz project (EIR file number IIA MT0655-134-2018) and Fénix (EIR file number M2650-2017). In Argentina, EIRs serve as the main official source of information concerning environmental and social issues in mining projects.

Only battery-grade Li₂CO₃ was considered to estimate the WF for both projects. LiCl produced by MdA was excluded for two reasons. First, the EIR (file number M2650-2017) clearly states that freshwater involved in its production is obtained from water sources around the city of General Güemes, 500 km away from the Fénix camp. Second, as noted by Díaz Paz et al. (2023) [3], concerns about water consumption in lithium extraction only relate to the Puna region, as also pointed out by Marconi & Clark (2022) [5] and Escosteguy et al. (2024) [77].

The WF of Li_2_CO_3_ battery-grade production (m^3^/ton) was estimated following the methodology proposed by Hoekstra et al. (2011) [28] in the Water Footprint Assessment Manual. The ${WF}_{blue}$ was estimated only about freshwater by dividing the water inputs and outputs from the production process (m^3^) over the year by the amount of Li_2_CO_3_ battery-grade production in Olaroz and Fénix project (ton) (equation (1)). Brine consumption (BC) was estimated separately:

(1) $${WF}_{blue}=\frac{{Water}_{inflow}-{Water}_{outflow}}{Production}$$

In this study, we assume ${Water}_{outflow}$ to be zero, based on two criteria: (1) as described in Section 2.3, the outflow consists of wastewater that does not have the same physical and chemical characteristics as the initial water inflows, and (2) freshwater does not return to its original basin -at least not immediately-so it is in fact removed from the hydrological system and it is no longer available [28]. It is important to note that wastewater from both projects consists mainly of recycled brine depleted of Li^+^ ions and recycled wash water. Approximately 90 % of that wastewater evaporates into the atmosphere, and only about 10 % is prone to infiltration [76].

The ${\;WF}_{green}$ is zero because there is no water consumption related to rainwater harvesting in any phases of the production processes. As mentioned in section 2.3., the case studies use only groundwater and surface water (WF_blue_).

The ${\;WF}_{gray}$ (m^3^/ton) was estimated by a mass balance of pollutants (m^3^/month) divided by the amount of Li_2_CO_3_ battery-grade production in Olaroz and Fénix (ton):

(2) $${WF}_{gray}=\left[\frac{Q_{e}\times \left(C_{Qe}-C_{\max}\right)}{\left(C_{\max}-C_{nat}\right)}\right]\times 12/Production$$

Here $Q_{e}$ (m^3^/month) is the effluent volume generated at the facilities of each project; $C_{Qe}$ (mg/L) the pollutant concentration; $C_{\max}$ (mg/L) the maximum allowable concentration of the pollutant; and $C_{nat}$ (mg/L) the concentration of the pollutant in the receiving water if the interferences of human activities are eliminated. The estimated WF_gray_ corresponds exclusively to the proportion of domestic effluents generated in the workers' camp. Due to the information available in the EIRs, this study could not identify the WF_gray_ associated with industrial effluents from the Li_2_CO_3_ battery-grade extraction processes.

### 2.5 Brine consumption estimation

Despite the high concentration of soluble ions, the chemistry and physical characteristics of brine closely resemble those of freshwater [78]. Furthermore, during the evaporative concentration phase, “pure water molecules” from the aqueous phase of brine are lost to the atmosphere [79]. Therefore, although brine consumption in lithium extraction does not fall within the WF concept, we consider it relevant to include this component in the analysis for the following reasons:

1. From a hydrological perspective, the understanding of aquifer hydrodynamics (both freshwater and brine) in the Puna region is limited [80,81]. Given this situation, predicting the potential environmental impacts of brine extraction on freshwater aquifers carries significant uncertainty.
2. The reduction in hydraulic head in brine aquifers caused by pumping could lead to the salinization of freshwater aquifers [72,82]. In terms of WF, according to Jamshidi (2019) [83], from an ecological perspective, this salinization would involve additional freshwater consumption to compensate for the hydraulic head in brine aquifers.
3. Salt flats, like freshwater aquifers, wetlands, native flora and fauna, are integral components of a unique and complex ecosystem known as the “Puna wetlands” [84,85]. The alteration of these ecosystems due to lithium extraction affects biodiversity and the provision of ecosystem services, which are linked to both brine and freshwater extraction [1,67].
4. The social dimension of the territories affected by lithium extraction shows that salt flats are part of the identity and culture of local communities living in the Argentine Puna [16]. In this regard, Blair et al. (2024) [2] argues that a hydrosocial dimension shapes local ways of life, often excluded from the life cycle assessment (LCA) intended to study water consumption in lithium extraction. From this hydrosocial perspective, it is necessary to study the whole water consumption of lithium extraction (both freshwater and brine), not just freshwater consumption [86].

Brine Consumption (BC) was estimated by dividing the brine inputs and outputs from the production process (m^3^) over the year by the amount of Li_2_CO_3_ battery-grade production in Olaroz and Fénix (ton) (equation (3)):

(3) $$BC=\frac{{Brine}_{inflow}-{Brine}_{outflow}}{Production}$$

Based on the WF_blue_ criteria described above, ${Brine}_{outflow}$ was assumed to be zero. Given that the evaporative concentration phase typically spans 12–18 months [22], we used the volume of brine pumped in 2020 to estimate the BC consumed in the 2021 production cycle.

### 2.6 Blue water intensity estimation

To compare the WF_blue_ of the lithium extraction case studies with the blue water availability at the hydrological unit level, we estimated the blue water intensity (${WI}_{blue}$) in the basin. This indicator represents an adaptation of the Blue Water Scarcity (WS_blue_), a metric proposed by the WF assessment methodology [28]. WI_blue_ only considers the WF_blue_ of the selected case studies. We define WIblue as the ratio between the WF_blue_ of each lithium extraction project and the availability of blue water in the environment.

(4) WIblue=WFblueWAblue

Where ${WF}_{blue}$ is the blue water footprint (m^3^/y) at each project in 2021; and ${WA}_{blue}$ (m^3^/y) is the blue water availability in the basin -defined as the natural blue water stocks (water stored in rivers, lakes, and aquifers) (${WS}_{nat}$) (m^3^/y)- minus the theoretical environmental flow requirement (EFR) (m^3^/y) (equation (5)). EFR is defined as the quantity of water flows required to sustain wetlands and human livelihoods. Theoretical EFR was estimated as 20 % of the ${WS}_{nat}$ [28]:

(5) $${WA}_{blue}={WS}_{nat}-EFR$$

### 2.7 Population equivalent

Population equivalent (PE, inhabitants) was estimated by dividing the ${WF}_{blue}$ (m^3^/y) at each lithium extraction project for the 2021 production cycle by theoretical water consumption of the local population (HWC) (m^3^/y) [87]:

(6) PE=WFblueHWC

The theoretical HWC (m^3^/y) was estimated with the following equation:

(7) HWC=V∗N

Where V (m^3^/y) is the volume of water consumed per capita and N (inhabitants) is the population in the area considered.

A detailed description of the methodology and calculations performed in this study is provided in the supplementary material.

## 3 Results and discussion

### 3.1 Water footprint

The total WF for Olaroz in 2021 was estimated at 51.0 m^3^/ton. This includes WF_blue_ (46.7 m^3^/ton) and WF_gray_ (4.3 m^3^/ton) components. In Fénix, the total WF in 2021 was estimated at 135.5 m^3^/ton, including only WF_blue_. From Table 1 it can be calculated that although battery-grade Li_2_CO_3_ production was 1.5 times higher at Fénix than at Olaroz, the total WF per unit of product was 2.7 times greater at Fénix. In terms of annual WF -based on 2021 production levels-the total WF of Fénix was 4 times larger than that of Olaroz.

**Table 1:** Comparative WF and BC in the case studies.

Project	Production	Total WF		Total BC	
	(ton/y)	m^3^/ton	m^3^/y	m^3^/ton	m^3^/y
Olaroz	12,611	51.0	643,439	537.4	6,777,192
Fénix	19,000	135.5	2,574,107	319.6	6,072,330
Fénix – Olaroz	6,389	84.5	1,930,667	−217.8	−704,862
Fénix/Olaroz	1.5	2.7	4.0	0.6	0.9

The technical feasibility of a lithium extraction project in salt flats is highly dependent on access to freshwater [88]. This strong dependence on freshwater access is linked to the Li^+^ ion recovery process from brine, which cannot occur without freshwater consumption [89]. Meng et al. (2019) [90] have reported that freshwater consumption is conditioned by and dependent on the characteristics of the brine, such as the concentration of residual ions (e.g. Na^+^, K^+^, Mg^2+^, SO_4_^2−^) and the natural concentration of Li^+^ ions. In that regard, the brine from Hombre Muerto salt flat (Fénix) is characterized by a lower concentration of SO_4_^2−^ with a higher prevalence of Na^+^ and Mg^2+^ while the brine from Olaroz salt flat has a higher concentration of SO_4_^2−^ with a greater predominance of Na^+^ and K^+^ [91]. The average Li^+^ ion concentration in the brine was determined at 628 ppm (Hombre Muerto salt flat) and 841 ppm (Olaroz salt flat) [92,93]. Water consumption also depends on technical aspects such as the production level and the purity of the product [94]. Although the purity of battery-grade Li_2_CO_3_ was the same (97.7 % LCE) [6,7], the total WF at Fénix is considerably higher than at Olaroz (see Table 1).

Our results are in line with the findings of Halkes et al. (2024) [95], showing that DLE is more intensive in freshwater consumption. Vera et al. (2023) [25] argue that DLE technologies require sustainable freshwater supply alternatives. Local freshwater uses could be impacted by the growing water appropriation driven by the expansion of lithium extraction, an industry expected to accelerate further due to recent shifts in Argentina's national environmental, corporate, and foreign investment policies [96].

Baseline information from EIRs of both Olaroz and Fénix does not adequately report the type, volume, and characteristics of the wastewater discharged to the environment in order to calculate the WF_gray_. Documents assessed show that effluents consist of a mixture of brine with low concentrations of Li ^+^ ions (depleted brine) and wash water, pumped through a pipeline system and disposed on the surface of the salt flat for infiltration. However, as indicated above, the aqueous phase of these effluents is mainly lost to the atmosphere through evaporation [76]. A potential source of WF could be associated with the volume of groundwater involved in diluting the brine to compensate for pumping [66]. In this analysis, the connection between the aquifers determines the WF component involved: if brine infiltrates a freshwater aquifer, contamination of the freshwater would occur, which could be estimated using the WF_gray_. Conversely, if freshwater infiltrates a brine aquifer, it would represent a form of blue water consumption, which could be estimated using the WF_blue_. The ISO 14046 standard defines freshwater consumption as water that is lost to the basin of origin due to evaporation, incorporation into products, or discharge into other basins or the sea. Thus, infiltration into a brine aquifer is analogous to discharge into the sea. However, it was not possible to advance this analysis in this study due to insufficient data provided in the EIRs. The WF_gray_ for Olaroz reported above is related to wastewater from the camp, which is treated and discharged into an infiltration field. It represents the water needed to assimilate the concentration of nitrates discharged with the wastewater. The WF_gray_ was not estimated in Fénix because the wastewater associated with the camp was not reported in the EIR.

### 3.2 Brine consumption

BC for Olaroz and Fénix in 2021 was 537.4 m^3^/ton and 319.6 m^3^/ton, respectively. The results shown in Table 1 indicate that Olaroz was more intensive in brine consumption than Fénix. Per unit of product, Olaroz consumed 217.8 m^3^ more brine than Fénix and produced 6,389 ton less battery-grade Li_2_CO_3_ of the same purity. There is a clear technological advantage of DLE at Fénix over conventional evaporative at Olaroz, with 1.5 times higher production and 0.6 lower brine consumption.

Our WF and BC results indicate that studying water consumption in lithium extraction requires a holistic perspective that considers the entire hydrological system, as focusing solely on freshwater consumption introduces a bias related to the system boundary definition. When considering only WF, Fénix appears more water-intensive; however, by incorporating both components of the hydrological system, our results show that while Fénix has a WF per unit of product 2.7 times higher than Olaroz, its total annual water use in 2021 was less intensive than that of Olaroz.

### 3.3 Blue water footprint in perspective

From Table 2, it can be seen that at Olaroz, WF_blue_ accounted for 30 % of the WA_blue_, whereas at Fénix, consumption was 10 %. Due to limited monthly information, these estimations were based on annual production data (for the 2021cycle), although Hoekstra et al. (2011) [28] recommends analyzing monthly values to detect variations in WF_blue_ and WA_blue_. Additionally, our results include only water consumption from two projects, excluding other lithium extraction projects and local productive activities in the basins. Brine consumption, a significant component of water use in salt flat lithium extraction, was also not included. In both cases, only the availability of blue groundwater was considered.

**Table 2:** Comparative WI_blue_ and PE in the case studies.

Project	WF_blue_ (m^3^/y)	WI_blue_		PE (inh)
		Value	Categories	
Olaroz	589,168	0.301	Significant intensity	32,283
Fénix	2,574,107	0.099	Moderate intensity	141,047

Our WI_blue_ results indicate that, although the WF_blue_ at Fénix is higher than at Olaroz, its effect on local blue water availability is less severe, placing it in a moderate WI_blue_ intensity category. It is important to note that these findings are not conclusive and require longer-term studies to clarify the local implications of water consumption, particularly considering that both projects are expanding their production capacity and that others lithium extraction projects are operating in both salt flats since 2023 [97]. However, these findings underscore the importance of contextualizing WF_blue_ values to assess the extent to which freshwater use in lithium extraction affects local water availability.

Our results also show that the WF_blue_ at Olaroz in 2021 was equivalent to 8 times the annual water consumption of the entire population of the Susques department (3,980 inhabitants). In contrast, the WF_blue_ at Fénix was equivalent to 70 times the annual water consumption of the population of the Antofagasta de la Sierra department (2,022 inhabitants).

The relative comparison between the amount of freshwater consumption involved in a particular production process to its equivalent in population water consumption has significant utility and importance in managing water resources [98]. In the specific context of the Puna region, this issue gains significance due to the conflicting interests and competing uses of freshwater between lithium mining companies and local communities [3]. According to the salinity classification proposed by Li et al. (2020) [99], the water consumed in the production processes at Olaroz and Fénix falls into the freshwater category. While the freshwater category does not immediately imply suitability for human consumption, Onyebuchi Okafor et al. (2022) [100] argue that the natural presence of freshwater sources (surface or groundwater) ensures the physical availability of a drinking water source. Conversely, the absence of freshwater sources leads to physical drinking water scarcity.

### 3.4 Discussion and limitations of this study

Our results indicate that freshwater availability is more of a constraint to DLE projects than conventional evaporation technologies. The current expansion of the production capacity of Fénix includes the construction of a new freshwater extraction system located 35 km from the project facilities [6]. Expanding DLE must involve identifying alternative water supply sources to reduce the consumption of available freshwater. Brine desalination could be a potential alternative source of freshwater for lithium extraction projects [23]. However, the extraction of too much brine can promote the migration of groundwater towards salt flats [68]. In this regard, greater knowledge of local hydrological systems is essential for accurately assessing the environmental impact of lithium mining. Our results also suggest that there might be unforeseen competition between faster lithium extraction and freshwater consumption.

In interpreting our results, it is essential to consider that the WF represents only the direct water consumption associated with lithium extraction. From a global water resource perspective, future research should delve deeper into the WF linked to indirect water consumption in lithium extraction, such as the WF associated with electricity consumption. Standardized estimates suggest that the WF of electricity consumption is approximately 2,000 m^3^/TJ, with variations depending on the energy source powering the electrical grid [101]. Based on this evidence, it is likely that the indirect WF of lithium extraction exceeds its direct WF. However, it is crucial to consider the spatial scale of the direct WF, which occurs within an environmental, ecological, and social context highly dependent on the limited local availability of water under current climatic conditions. While, as highlighted by the International Lithium Association (ILiA), understanding the global WF of lithium-derived products and their impact on the electric vehicle and lithium-ion battery markets is important [34], this global perspective should not overshadow or obscure local demands. In regions where lithium is extracted, concerns are not focused on water consumption throughout the entire life cycle of lithium but rather on the uncertainty surrounding future water availability in territories currently under exploitation.Reporting freshwater consumption in lithium extraction using the theoretical WF framework allowed us to identify water consumption according to the type of water source (WF_blue_ or WF_gray_) and estimate the WI_blue_ -a useful tool to contextualize the water consumption involved in human activities. The WF also allows for standardization of reference values according to the type of technology used and the level of production achieved. It enables relative comparison of water consumption for different activities in the same water catchment. WF could therefore be used as a key performance indicator for the technical, environmental, and social assessment of lithium extraction projects. Estimating the WF of lithium extraction projects becomes more relevant when local concerns about water provision are high. Unfortunately, water consumption data reported by mining companies in their sustainability reports often lack context and, at times, are ambiguous, which can lead to misinterpretations.

Despite their limitations, EIRs still represent a viable primary data source for advancing comparative studies of water consumption in lithium extraction, being the only official documents used to government offices to make decisions. Argentine government agencies do not collect primary data nor publish reports related to environmental variables relevant to lithium extraction projects [21], and therefore EIRs are often the only source of information available to researchers and local communities to assess the impacts of lithium extraction. It would be useful for these official documents to include minimum and standardized data regarding water consumption. We found discrepancies in the type and among of information available in the different EIRs. The EIR of Olaroz provides a comprehensive description of water consumption, discriminated by volume, percentage, and production process phases. In contrast, the EIR of Fénix provides data only on the volume of water consumption. These differences are reflected in the WF results of this study, as the EIR of Fénix lacks the necessary information to identify, for instance, WF_gray_. At the same time, information on water consumption in the EIRs, when available, is frequently too technical for civil society and decision-makers to understand [102] or for small, usually poor local governments to audit or verify the information provided [103].

The original definition of the WF refers only to freshwater [28]. However, in the case of lithium extraction, we believe that it is also necessary to include brine consumption. When brine pumped from salt flats enter the evaporative concentration phase, a loss of the aqueous phase occurs due to solar evaporation. While it might be assumed that the evaporated water returns through precipitation as part of the hydrological cycle, this does not occur in the Puna region, because extraction rates significantly exceed annual water input from precipitation [1,67]. As indicated above, separating groundwater from brines in Andean salt flats is complex because they are hydrologically connected. This is particularly relevant when the rates of brine and groundwater extraction are important, as in lithium extraction. This inextricable connection means that extracting brines from salt flats competes, at least indirectly, with other local consumptive uses of freshwater.

Our WI_blue_ results indicate that the implications of water consumption in lithium extraction vary depending on the geographic context. At Fénix, although freshwater consumption was higher than at Olaroz, its impact was moderate because of a greater local blue water availability. This suggests that, in terms of water consumption, the environmental performance of a technology depends also on geographic factors, an aspect often overlooked when assessing the advantages of new technologies.

On a global scale, the supply of drinking water necessarily involves different forms of pre-treatment of freshwater sources, which can be achieved with cost-effective technologies [49]. Defining freshwater as “industrial use water not suitable for human consumption” [7] is a misleading discourse by mining companies that tries to hide the fact that lithium extraction does compete with consumptive uses at the local scale (SMeH, 2023) [8]. According to Khondoker et al. (2023) [104], the supply of water for human consumption in any industry should be the primary consideration in water resource management.

Estimating the PE provides an easy-to-understand and context-based metric of the scale of water use in industrial activities [87]. This indicator highlights (and arguably even exacerbates) potential competition for water resources between local actors and mining activities. It could help frame the water debate and hopefully also contribute to boosting water conservation efforts [105].

Lithium extraction also disrupts ecosystem services associated with salt flats and surrounding wetlands. The opportunity cost of this disruption has been described and analyzed from a social [21,103,106] and environmental perspective [65,107,108]. Cumulative environmental and social assessments are needed in basins where there are several lithium extraction projects, in order to prevent critical impacts on local water resources. Grassroots and indigenous movements have highlighted the increasing environmental costs of lithium extraction and denounced mining companies for turning sites into “sacrifice zones” [109,110]. Competition for water resources among mining companies, local communities, and the environment is not entirely fair, with national, regional, and local governments openly promoting this activity with all sorts of benefits that include tax exemptions, subsidies, maintenance of roads, new natural gas pipes, and infrastructure that is often not available to local populations [77].

## 4 Conclusions

The WF of the Olaroz and Fénix projects was estimated for battery-grade Li_2_CO_3_ based on data from EIRs. The total WF was 51.0 m^3^/ton and 135.5 m^3^/ton at Olaroz and Fénix, respectively. Differences between projects are based mainly on the technological aspects (DLE versus conventional evaporative techniques). Per unit of product, WF in Fénix was 2.7 times greater than in Olaroz. On the other hand, BC per unit of product was 0.6 times lower in Fénix than in Olaroz despite the fact that Fénix produces 1.5 times more than Olaroz. This efficiency does not translate into reduced freshwater consumption.

WI_blue_ and PE are useful indicators to contextualize the impacts of lithium extraction projects on local basins and the livelihoods of local communities associated with the salt flats. Our WI_blue_ results indicate that the implications of water consumption in lithium extraction varies depending on the geographic context. At Fénix, although freshwater consumption was higher than at Olaroz, its impact was moderated by greater local WA_blue_.

The WF_blue_ at Olaroz was equivalent to 8 times the annual water consumption of the population in the Susques department. In contrast, the WF_blue_ at Fénix was equivalent to 70 times the annual water consumption of the population in the Antofagasta de la Sierra department. By emphasizing the potential or hypothetical social implications of industrial water use, estimation of the PE can drive more informed and equitable decision-making in water resource management.

A more in-depth knowledge of the hydrodynamics of surface and groundwater basins around lithium extraction sites seems necessary to prevent freshwater depletion and minimize potential cumulative impacts derived from different lithium extraction projects sharing the same basin. This could be the safest way to protect water resources, guarantee a more equitable mining activity, and avoid falling into the “hydro-fantasies” of the past.

In the assessed case studies, the technical data available in EIRs were sufficient for the estimation of the WF and the results obtained were in line with previous estimations. However, there are some limitations and challenges that need to be addressed, particularly related to the amount and precision of the information provided in those studies. It would be advisable for EIRs to include, for unconfined aquifers, a model of the infiltration rate of water from freshwater aquifers into brine aquifers induced by brine pumping. This is crucial because, if such water flow occurs, it would also contribute to the WF, associated with the volume of freshwater migrating into the brine aquifer - or, conversely, brine migrating into freshwater aquifers - to compensate for the loss of hydraulic head caused by successive pumping. By incorporating this analysis, the WF could be addressed in a more detailed, comprehensive, and context-specific manner, tailored to the unique conditions of lithium extraction in salt flats. This approach would not only enable a more accurate estimation of the impact of water consumption on local water resources but also provide valuable information to better understand salt flats and prevent potential environmental impacts from saline intrusion. All in all, we believe that the WF could be adopted as a benchmarking tool to compare lithium extraction technologies since it allows an easy and transparent way to compare mining initiatives and sheds some light on some of their most controversial social and environmental impacts.

## CRediT authorship contribution statement

**Walter Fernando Díaz Paz:** Writing – review & editing, Writing – original draft, Visualization, Methodology, Formal analysis, Data curation, Conceptualization. **Lucas Seghezzo:** Writing – review & editing, Supervision, Funding acquisition, Conceptualization. **Ariela Griselda Salas Barboza:** Writing – review & editing, Methodology, Formal analysis, Conceptualization. **Melisa Escosteguy:** Writing – review & editing, Conceptualization. **Paula Valentina Arias-Alvarado:** Methodology, Data curation. **Eduardo Kruse:** Supervision. **Marc Hufty:** Project administration, Funding acquisition. **Martín Alejandro Iribarnegaray:** Writing – review & editing, Supervision, Project administration, Funding acquisition, Formal analysis, Conceptualization.

## Data availability

All data included in this article is referenced in the supplementary material.

## Declaration of competing interest

The authors declare the following financial interests/personal relationships which may be considered as potential competing interests:Martin Alejandro Iribarnegaray reports financial support, article publishing charges, and travel were provided by 10.13039/501100002923National Scientific and Technical Research Council. Martin Alejandro Iribarnegaray reports financial support was provided by 10.13039/100000001Swiss National Science Foundation. Martin Alejandro Iribarnegaray reports financial support and article publishing charges were provided by Fundación Ambiente y Recursos Naturales. If there are other authors, they declare that they have no known competing financial interests or personal relationships that could have appeared to influence the work reported in this paper.

## Footnotes

1 Newspaper “Página 12”, 12 August 2023. Available at: https://www.pagina12.com.ar/577236-el-agua-y-el-litio-emblemas-de-la-transicion-energetica. Last accessed 25 January 2024.
