# Supplementary material for: The water footprint of lithium extraction technologies: Insights from environmental impact reports in Argentina's salt flats
Source: Heliyon. 2025 Feb 7;11(4):e42523. doi: 10.1016/j.heliyon.2025.e42523 (PMC11869023; doi:10.1016/j.heliyon.2025.e42523)
Supplement: Multimedia component 1 [file mmc1.docx]

**The Water Footprint of Lithium Extraction Technologies: Insights from Environmental Impact Reports in Argentina’s salt flats**

Walter Fernando Díaz Paz^a^, Lucas Seghezzo^a^, Ariela Griselda Salas Barboza^a^, Melisa Escosteguy^a^, Paula Valentina Arias-Alvarado^b^, Eduardo Kruse^c^, Marc Hufty^d^, Martín Alejandro Iribarnegaray^a*^

^a^ Consejo Nacional de Investigaciones Científicas y Técnicas (CONICET), Universidad Nacional de Salta (UNSa), Avenida Bolivia 5140, A4408FVY, Salta, Argentina

^b^ UNSa, Avenida Bolivia 5140, A4408FVY Salta, Argentina.

^c^ Centro de Investigaciones y Transferencia del Noroeste de la Provincia de Buenos Aires (CIT-NOBA), CONICET, Universidad Nacional de La Plata (UNLP), Casco Urbano, B1900, La Plata, Provincia de Buenos Aires, Argentina

^d^ Centre for International Environmental Studies, Geneva Graduate Institute, 2 Ch. Eugene-Rigot, CH1211, Geneva, Switzerland

^*^Corresponding author

Corresponding author email: [miribarnegaray@conicet.gov.ar](mailto:miribarnegaray@conicet.gov.ar)

**Supplementary material**

**Methodology**

1. **WF_blue_ estimation:**

**Table** 1 shows the freshwater inflows identified in each project. The 2021 yearly battery-grade Li_2_CO_3_ production at Olaroz and Fénix was 12,611 and 19,000 tons, respectively [1,2]. $Freshwater inflow$ (m^3^) was estimated by following the equation (1), see **Table 2**:

$$Freshwater inflow=\sum_{t=1}^{12} {Pumping}_{Freshwater}(m^{3}) (1)$$

Table 1. Monthly freshwater inflow identified in the case studies

| Month | **Olaroz^[[1]](#footnote-1)^** | **Fénix^[[2]](#footnote-2)^** |
| --- | --- | --- |
|  | Freshwater pumping (m^3^) | Freshwater pumping (m^3^) |
| January | 69,837.8 | 233,771.0 |
| February | 58,153.0 | 211,494.8 |
| March | 46,030.5 | 209,133.5 |
| April | 32,077.0 | 199,585.5 |
| May | 36,310.2 | 208,837.4 |
| June | 53,069.6 | 192,481.2 |
| July | 34,500.2 | 230,507.9 |
| August | 24,761.5 | 177,812.0 |
| September | 46,108.3 | 227,710.0 |
| October | 51,841.8 | 241,051.8 |
| November | 56,441.4 | 198,194.7 |
| December | 80,036.9 | 243,494.8 |

Table 2. Annual freshwater inflows estimated in the case studies

| **Item** | **Olaroz** | **Fénix** |
| --- | --- | --- |
| Freshwater inflow (m^3^) = ∑ Monthly pumping | 589,168.0 | 2,574,074.6 |

In addition to the freshwater volumes consumed in the production process, at Fénix we identified water consumption associated with an open-air reservoir, where freshwater is stored before entering the plant with losses due to evaporation. According to Hoekstra et al. (2011) [3], this consumption should be considered as a component of the WF_blue_ in the estimation. Although the climatic conditions in the Puna region result in high evaporation and transpiration rates year-round [4], previous studies have shown that lithium extraction facilities alter the natural environmental conditions. Weinberg (2023) [5] refers to these alterations collectively as the “off-sites” of lithium extraction:

- Stamp et al. (2012) [6] demonstrated that water consumption in lithium extraction (both brine and freshwater) affects biodiversity and compromises land use.
- Marazuela et al. (2019) [7] demonstrated that the pumping of freshwater and brine, combined with water loss in evaporation ponds, alters the natural evaporation rate on the surface of salt flats - an effect the authors termed “damping capacity.”

Based on the background cited previously, we consider it appropriate to include this type of water consumption in the WF_blue_ estimation.

The total volume of evaporated water ($EW$, m^3^) associated with the freshwater open-air reservoir at Fénix was estimated by following the equation (2). See **Table 3**

$$EW=\left( 10\times\sum_{t=1}^{365} ET \right)\times A (2)$$

The evaporation from the water surface ($ET$, see **Table 4** ) was estimated using the Penman-Monteith equation. The equation (3) was written by the Food and Agriculture Organization of the United Nations – FAO, paper N°56 [8]:

$$ET=\frac{0.408\times\left( R_{n}-G \right)+\gamma\times\frac{900}{T+273}\times u_{2}\times\left( e_{s}-e_{a} \right)}{\Delta+\gamma\times\left( 1+0.34\times u_{2} \right)} (3)$$

Where $ET$ is the evaporation from the water surface (mm/day); $R_{n}$net radiation (MJ/m^2^.day); $G$ the change in heat storage in the water body (MJ/m^2^.day); $\gamma$ the psychrometric constant (kPa/°C); $T$ air temperature at a height of 2 meters (°C); $u_{2}$wind speed at a height of 2 meters (m/s); $e_{s}$ the saturated vapour pressure at water temperature (kPa); $e_{a}$the vapour pressure at air temperature (kPa); $\Delta$ the slope of the temperature saturation water vapour curve at water temperature (kPa/°C).

The Penman-Monteith equation has been used to estimate ET in study areas where instrumental measurements are not available [9,10]. This equation is the result of an empirical development based on the physical principles of heat transfer and combines all the determining variables of the ET from an evaporative surface [8]. The equation provides an ET value which must be corrected by a crop coefficient in the case where the evaporative surface is covered by vegetation [11]. However, for open-water surfaces, the ET value obtained does not require correction [12]. Consequently, for this work, the Penman-Monteith equation is considered adequate for estimating ET from the Fénix freshwater reservoir.

The NASA geospatial database (<https://power.larc.nasa.gov>) was used to obtain data for the meteorological variables required by the Penman-Monteith equation.

Table 3. Evaporated water (EW) estimation

| **Item** | **Value** |
| --- | --- |
| Total ET=∑ET montly (mm) | 913.54 |
| Conversion factor | 10 |
| A (ha) | 0.0034941 |
| EW (m^3^/y) | 31.92 |

Table 4. ET estimation. The daily ET values have been expressed monthly

| **ET by Penman-Monteith equation (mm/month)** | | | | | | | | | | | |
| --- | --- | --- | --- | --- | --- | --- | --- | --- | --- | --- | --- |
| January | February | March | April | May | June | July | August | September | October | November | December |
| 129.53 | 108.73 | 91.38 | 65.53 | 22.73 | -1.02 | 11.31 | 30.76 | 83.46 | 102.82 | 135.07 | 133.22 |

The ${WF}_{blue}$ (m^3^/ton) was estimated by following the equation (4). See **Table 5**:

$${WF}_{blue}=\frac{{Water}_{inflow} - {Water}_{outflow}}{Production} (4)$$

In this study, we assume ${Water}_{outflow}$ to be zero, based on two criteria: (1) as described in Section 2.3, the outflow consists of wastewater that does not have the original physical and chemical characteristics as the initial water inflows, and (2) the freshwater does not return to its original basin - at least not immediately - so it is water that is removed from the hydrological system and no longer available for future local uses [3]. It is important to note that the wastewater from both projects consists mainly of recycled brine depleted of Li^+^ ions and recycled wash water. From this mixture, approximately 90% of the total wastewater volume evaporates into the atmosphere, and only about 10% is prone to infiltration [13].

Table 5. WF_blue_ (m^3^/ton) estimated in the case studies

| **Olaroz** | | |
| --- | --- | --- |
| Item | | WF_blue_ |
| Water inflow (m^3^/y) | 589,168.03 | 46.7 |
| Water outflow (m^3^/y) | 0 |  |
| Production (ton) | 12,611 |  |
| **Fénix** | | |
| Water inflow (m^3^/y) | 2,574,074.6 | 135.5 |
| EW (m^3^/y) | 31.92 |  |
| Water outflow (m^3^/y) | 0 |  |
| Production (ton) | 19,000 |  |

1. **WF_gray_ estimation:**

The ${WF}_{gray}$ (m^3^/ton) was estimated in the context of Olaroz facilities and only included the domestic wastewater. The methodology proposed by Hoekstra et al. (2011) [3] for estimating the WF_gray_ (Equation 5) provides sensitivity and scope for studies of a global scale but has limitations for application in focused case studies [14].

$${WF}_{gray}=\frac{L}{C_{max}-C_{nat}} (volume/time) (5)$$

In previous studies, some proposals for methodological adaptations to estimate the WF_gray_ in focused case studies have already been tested and validated [15,16,17]. Morera et al. (2016) [16] proposed an adaptation of Equation 6 based on the mass balance of the pollutant load at the discharge, where the WF_gray_ is defined as the minimum volume of water required (m^3^) to dilute the pollutant concentration ($Q_{e}$), given a maximum allowable concentration of the pollutant ($C_{max}$) and its natural concentration in water without human disturbance ($C_{nat}$) (Equation 6). In the context of this work, the methodological adaptation proposed by Morera et al. (2016) is considered appropriate.

$${WF}_{gray}=\left[ \frac{Q_{e}\times\left( C_{Qe}-C_{max} \right)}{\left( C_{max}-C_{nat} \right)} \right]\times12/ production (6)$$

Here $Q_{e}$ (m^3^/month) is the effluent volume generated at Olaroz project facilities, $C_{Qe}$ (mg/L) the pollutant concentration in the effluent; $C_{max}$ (mg/L) maximum allowable concentration of the pollutant; $C_{nat}$ (mg/L) the concentration of the pollutant in the receiving water if the interferences of human activities are eliminated.

Considering the chemical composition of domestic wastewater where nitrogen (N) is one of the most predominant pollutants [18,19]; and that in water without human disturbance, the presence of N is close to 0.01 mg/L [15]; added to the fact that the prevalence of nitrogen compounds in water resources triggers anthropogenic processes of eutrophication [20]. In this work, it is appropriate to adopt the N concentration as the effluent pollutant susceptible to dilution. **Table 6** shows the parameters used in equation (6) to estimate the indirect WF_gray_. The effluent volume of domestic wastewater ($Q_{e}$) has been taken from the EIR file number IIA MT0655-134-2018; the values for, $C_{Qe}$ and $C_{max}$assume a pre-treatment of the effluent before discharge, in the literature we can find different values suggested for these parameters [21]. For this study, we have taken the values provided by the WF_gray_ guidelines, which are based on the guidelines for the protection of aquatic life as proposed by the Canadian Council of Ministers of the Environment [22]. Following the WF_gray_ guidelines from the Water Footprint Network for water bodies without human disturbance $C_{nat}$ have been considered zero [15].

Table 6. WF_gray_ estimation at Olaroz project

| **WF_gray_ (m^3^/y)** | | |
| --- | --- | --- |
| Item | | WF_gray_ |
| Q_e_ (m^3^/month) | 641.0 | 54,271.2 |
| C_Qe_ (mg/L) | 2.9 |  |
| C_max_ (mg/L) | 0.36 |  |
| C_nat_ (mg/L) | 0 |  |
| Conversion factor | 12 |  |
| **WF_gray_ (m^3^/ton)** | | |
| Production (ton) | 12,611 | 4.3 |

1. **Total WF estimation:**

The total WF involved in the cycle production during a period of time is defined as the following:

$$Total WF=\sum{WF}_{Blue}+{WF}_{green}+{WF}_{gray} (7)$$

Considering the specific context of the case studies, **Table 7** presents the total WF (m^3^/ton) in the battery-grade Li_2_CO_3_ production in the 2021 year, which is defined as follows equation 8 (at Olaroz), equation 9 (at Fénix):

$$Total WF=\sum{WF}_{blue}+{WF}_{gray} (8)$$

$$Total WF=\sum{WF}_{blue} (9)$$

Table 7. Total WF estimation in the specific context of the case studies

| **Olaroz** | |
| --- | --- |
| WF_blue_ | 46.7 |
| WF_gray_ | 4.3 |
| Total WF (m^3^/ton) | 51.0 |
| **Fénix** | |
| WF_blue_ | 135.5 |
| Total WF (m^3^/ton) | 135.5 |

1. **Brine consumption estimation:**

$Brine inflow$ (m^3^) were estimated by following the equation (10). **Table 8** shows the monthly brine inflows identified in each project and **Table 9** shows the annual brine inflows estimated.

$$Brine inflow=\sum_{t=1}^{12} {Pumping}_{Brine}(m^{3}) (10)$$

Table 8. Monthly brine inflows in the case studies

| Month | **Olaroz^[[3]](#footnote-3)^** | **Fénix^[[4]](#footnote-4)^** |
| --- | --- | --- |
|  | Brine pumping (m^3^) | Brine pumping (m^3^) |
| January | 674,474.0 | 581,993.0 |
| February | 524,084.5 | 526,096.0 |
| March | 373,695.0 | 380,936.0 |
| April | 480,911.8 | 464,493.0 |
| May | 711,083.3 | 564,426.0 |
| June | 614,431.0 | 543,060.0 |
| July | 430,270.1 | 544,937.0 |
| August | 246,698.2 | 384,632.0 |
| September | 659,436.9 | 478,696.0 |
| October | 540,662.0 | 510,470.0 |
| November | 564,865.0 | 507,337.0 |
| December | 956,580.6 | 585,254.0 |

Table 9. Annual brine inflow in the case studies

| **Item** | **Olaroz** | **Fénix** |
| --- | --- | --- |
| Brine inflow (m^3^) = ∑ Monthly pumping | 6,777,192.5 | 6,072,330.0 |

Brine consumption ($BC$, m^3^/ton) was estimated by dividing the brine inputs and outputs from the production process (m^3^) over the year by the amount of Li_2_CO_3_ battery grade production in Olaroz and Fénix project (ton) (equation 11). Based on the WF_blue_ criteria described above, ${Brine}_{outflow}$was assumed to be zero. See **Table 10**:

$$BC=\frac{{Brine}_{inflow}- {Brine}_{outflow}}{Production} (11)$$

Table 10. BC (m^3^/ton) estimated in the case studies

| **Olaroz** | | |
| --- | --- | --- |
| Item | | BC |
| Brine inflow (m^3^/y) | 6,777,192.5 | 537.4 |
| Brine outflow (m^3^/y) | 0 |  |
| Production (ton) | 12,611 |  |
| **Fénix** | | |
| Brine inflow (m^3^/y) | 6,072,330.0 | 319.6 |
| Water outflow (m^3^/y) | 0 |  |
| Production (ton) | 19,000 |  |

1. **Blue water intensity estimation:**

Blue water intensity (${WI}_{blue}$, see **Table 11**) is defined as the following:

$${WI}_{blue}=\frac{{WF}_{blue}}{{WA}_{blue}} (12)$$

Where ${WF}_{blue}$ is the blue water footprint (m^3^/y) at each lithium mining project in the 2021production cycle; and ${WA}_{blue}$ (m^3^/y) is the blue water availability in the basin defined as the natural blue water stocks (the water volumes stored in rivers, lakes, and groundwater aquifers) (${WS}_{nat}$) (m^3^/y) minus the theoretical environmental flow requirement ($EFR$) (m^3^/y) (**Table 12**):

$${WA}_{blue}={WS}_{nat}-EFR (13)$$

Due to the lack of empirical data on environmental flow requirements in the Puna region, this study adopted the theoretical $\boldsymbol{EFR}$ suggested by Hoekstra et al. (2011) [3], setting EFR at 20% of $\boldsymbol{WS}_{\boldsymbol{nat}}$. The primary source of information used to obtain the $\boldsymbol{WS}_{\boldsymbol{nat}}$ values was the technical documents provided by the Mining and Hydrocarbons Agency of Jujuy and the Provincial Direction of Environmental Mining Management of Catamarca: (1) “Balance hidrológico y modelo conceptual e hidrogeológico de superficie de la cuenca Olaroz-Cauchari” [24], (2) “Estudio hidrogeológico Cuenca Río de los Patos – Salar del Hombre Muerto – Tomo I” [24], and (3) “Estudio hidrogeológico Cuenca Río de los Patos – Salar del Hombre Muerto – Segunda etapa. Informe Final” [25]. In estimating WS_nat_, only the sub-basin from which freshwater is extracted for the lithium mining projects was considered: Olaroz (Archibarca Sub-basin) and Fénix (Trapiche Sub-basin).

Table 11. WI_blue_ estimation in the case studies

| **Olaroz** | |
| --- | --- |
| WF_blue_ (m^3^/y) | 589,168.03 |
| WA_blue_ (m^3^/y) | 1,956,493.44 |
| WI_blue_ | 0.301 |
| **Fénix** | |
| WF_blue_ (m^3^/y) | 2,574,106.52 |
| WA_blue_ (m^3^/y) | 26,102,221.1 |
| WI_blue_ | 0.099 |

Table 12. WA_blue_ estimation in the case studies

| **Olaroz** | |
| --- | --- |
| WS_nat_ (L/seg) | 77.55 |
| WS_nat_ (m^3^/y) | 2,445,616.8 |
| EFR (m^3^/y) | 489,123.36 |
| WA_blue_ (m^3^/y) | 1,956,493.44 |
| **Fénix** | |
| WS_nat_ (L/seg) | 1,034.62 |
| WS_nat_ (m^3^/y) | 32,627,776.3 |
| EFR (m^3^/y) | 6,525,555.26 |
| WA_blue_ (m^3^/y) | 26,102,221.1 |

1. **Population equivalent estimation:**

Population equivalent ($PE$, see **Table 13**) is a dimensionless ratio between the freshwater consumed in the battery-grade Li_2_CO_3_ production at Olaroz and Fénix respectively (${WF}_{blue}$, m^3^/y), and the theoretical water consumption by local population ($HWC$) (equation 14):

$PE=\frac{{WF}_{blue}}{HWC} (14)$

Table 13. PE estimation in the case studies

| Item | **Olaroz** | **Fénix** |
| --- | --- | --- |
| WF_blue_ (m^3^/y) | 589,168.0 | 2,574,106.5 |
| HWC (m^3^/y) | 72,635.0 | 36,901.5 |
| PE | 8.0 | 70.0 |

PE shows in perspective the magnitude of freshwater consumption in the battery-grade Li_2_CO_3_ production in this region where the existence of water is climatically limited [26]. **Table 14** presents the ${WF}_{blue}$ (m^3^/y) estimation in the case studies.

Table 14. Annual WF_blue_ estimation in case studies

| Item | **Olaroz** | **Fénix** |
| --- | --- | --- |
| WF_blue_ (m^3^/ton) | 46.7 | 135.5 |
| Production in 2021 (ton/y) | 12,611 | 19,000 |
| WF_blue_ (m^3^/y) | 589,168.0 | 2,574,106.5 |

The theoretical $HWC$ (m^3^/y) was estimated by following the equation 15:

$$HWC=V\times N (15)$$

$V$ (m^3^/y) is the volume of water consumed per capita and $N$ (inhabitant) is the population in the area considered. Due to the lack of empirical data on water consumption in the population of the Puna region, this work adopted the value determined by the World Health Organization – WHO: 50 L/day per inhabitant. This value is based on a survey of 97% of the territory of Latin America, including the arid regions of Argentina, Bolivia, Chile, and Brazil [27]. According to this report, in regions such as the Puna, daily per capita water consumption is estimated as follows: 5 L/day for drinking; 20 L/day for personal hygiene; 15 L/day for food preparation and utensil washing; 10 L/day for other uses (e.g. household cleaning and laundry).

**Table 15** shows the estimated $HWC$, for the $V$ (m^3^/y.inhabitant) estimation has been considered the following equivalence (1m^3^=1,000 L) and (365days = 1year). The National Institute of Statistics and Census of Argentina - INDEC database was used to obtain the number of inhabitants ($N$) (<https://censo.gob.ar/>). To determine the $N$ value, the total population of the municipal departments defined as the area of influence of each lithium project was considered: Susques department (Olaroz Project) and Antofagasta de la Sierra department (Fénix Project).

Table 15. HWC estimation in the case studies

| Item | **Olaroz** | **Fénix** |
| --- | --- | --- |
| V (L/day.inhabitant) | 50 | 50 |
| Conversion factor | 1,000 | 1,000 |
| V (m^3^/day.inhabitant) | 0.05 | 0.05 |
| V (m^3^/y.inhabitant) | 18.25 | 18.25 |
| N (Inhabitants) | 3,980 | 2,022 |
| HWC (m^3^/y) | 72,635.0 | 36,901.5 |

**References**

[1] Livent Corp. Informe de Sustentabilidad “Crecer responsablemente”, 2021. <https://livent.com/wp-content/uploads/2022/10/Livent_2021SustainabilityReport-Spanish.pdf>.

[2] Orocobre. Sustainability Report 2021, 2021. <https://www.datocms-assets.com/53992/1638320314-orocobre-sustainability-report-2021-final-web.pdf>.

[3] A. Y. Hoekstra., A. K. Chapagain., M. Aldaya., & M.M. Mekonnen, The Water Footprint Assessment Manual Setting the Global Standard, First edition, *Earthscan, London, The United Kingdom, 2011*. <https://documents1.worldbank.org/curated/en/962651468332944887/pdf/626280PUB0The000Box0361488B0PUBLIC0.pdf>.

[4] R. García, E. Kruse, R. Etcheverry, M. Tessone, & P. Moreira, Características hidrogeológicas de los salares en la Puna Argentina. In Visintin, A., Etcheverry, R., & Rendtorff, N. (Ed.), El litio en la Argentina: visiones y aportes multidisciplinarios desde la UNLP., Universidad Nacional de La Plata, La Plata, Argentina, 2020, 45–54. <https://libros.unlp.edu.ar/index.php/unlp/catalog/view/1671/1650/5377-1>.

[5] Weinberg, M. The off-sites of lithium production in the Atacama Desert. *The Extractive Industries and Society*, *15* (2023) 101309. <https://doi.org/10.1016/j.exis.2023.101309>.

[6] Stamp, A., Lang, D. J., & Wäger, P. A. Environmental impacts of a transition toward e-mobility: the present and future role of lithium carbonate production. *Journal of Cleaner Production*, *23*(1) (2012) 104-112. <https://doi.org/10.1016/j.jclepro.2011.10.026>.

[7] Marazuela, M. A., Vázquez-Suñé, E., Ayora, C., García-Gil, A., & Palma, T. The effect of brine pumping on the natural hydrodynamics of the Salar de Atacama: The damping capacity of salt flats. *Science of the Total Environment*, *654* (2019) 1118-1131. <https://doi.org/10.1016/j.scitotenv.2018.11.196>.

[8] R.G. Allen., L.S. Pereira., D. Raes., & M. Smith, Food and Agriculture Organization of the United Nations (FAO): Irrigation and Drainage Paper N°56, 1998.

[9] Mekonnen, M. M., & Hoekstra, A. Y. The blue water footprint of electricity from hydropower. *Hydrology and Earth System Sciences*, *16*(1) (2012) 179–187. <https://doi.org/10.5194/hess-16-179-2012>.

[10] Zotarelli, L., Dukes, M. D., Romero, C. C., Migliaccio, K. W., & Morgan, K. T. Step by Step Calculation of the Penman-Monteith Evapotranspiration (FAO-56 Method). Institute of Food and Agricultural Sciences, University of Florida, 2013. <https://www.agraria.unirc.it/documentazione/materiale_didattico/1462_2016_412_24509.pdf>.

[11] Moran, M. S., Rahman, A. F., Washburne, J. C., Goodrich, D. C., Weltz, M. A., & Kustas, W. P. Combining the Penman-Monteith equation with measurements of surface temperature and reflectance to estimate evaporation rates of semiarid grassland. *Agricultural and Forest Meteorology*, *80*(2–4) (1996) 87–109. <https://doi.org/10.1016/0168-1923(95)02292-9>.

[12] Monteith, J. L. Evaporation and surface temperature. *Quarterly Journal of the Royal Meteorological Society. Royal Meteorological Society (Great Britain)*, *107*(451), (1981) 1–27. <https://doi.org/10.1256/smsqj.45101>.

[13] T. Kadri, Water infiltration in salt land in Nagekeo Flores, Indonesia. In Mohammed, B. S., Shafiq, N., Kutty, S. R., Mohammed, H., & Balogun, A. L. (Ed.), ICCOEE2020.ICCOEE 2021. *Lecture Notes in Civil Engineering, 132,* Springer Singapore (2021)74–80. <https://doi.org/10.1007/978-981-33-6311-3_9>.

[14] Mekonnen, M. M., & Hoekstra, A. Y. Global gray water footprint and water pollution levels related to anthropogenic nitrogen loads to fresh water. *Environmental Science & Technology*, *49*(21) (2015) 12860–12868. <https://doi.org/10.1021/acs.est.5b03191>.

[15] N.A. Franke., H. Boyacioglu., & A. Y. Hoekstra, Grey water footprint accounting: Tier 1 supporting guidelines. Value of water research report series N°65, *UNESCO-IHE*, *65, Delft, the Netherlands, 2013*. <https://ris.utwente.nl/ws/portalfiles/portal/5141740/Report65-GreyWaterFootprint-Guidelines.pdf>.

[16] Morera, S., Corominas, L., Poch, M., Aldaya, M. M., & Comas, J. Water footprint assessment in wastewater treatment plants. *Journal of Cleaner Production*, *112*, (2016) 4741–4748. <https://doi.org/10.1016/j.jclepro.2015.05.102>.

[17] Jamshidi, S. An approach to develop grey water footprint accounting. *Ecological Indicators*, *106* (2019), 105477. <https://doi.org/10.1016/j.ecolind.2019.105477>.

[18] Campos, H. M., & Von Sperling, M. Estimation of domestic wastewater characteristics in a developing country based on socio-economic variables. *Water Science and Technology: A Journal of the International Association on Water Pollution Research*, *34*(3–4) (1996) 71–77. <https://doi.org/10.2166/wst.1996.0418>.

[19] Van Drecht, G., Bouwman, A. F., Harrison, J., & Knoop, J. M. Global nitrogen and phosphate in urban wastewater for the period 1970 to 2050. *Global Biogeochemical Cycles*, *23*(4) (2009). <https://doi.org/10.1029/2009gb003458>.

[20] Serrano, L., Reina, M., Quintana, X. D., Romo, S., Olmo, C., Soria, J. M., Blanco, S., Fernández-Aláez, C., Fernández-Aláez, M., Caria, M. C., Bagella, S., Kalettka, T., & Pätzig, M. A new tool for the assessment of severe anthropogenic eutrophication in small shallow water bodies. *Ecological Indicators*, *76* (2017) 324–334. <https://doi.org/10.1016/j.ecolind.2017.01.034>.

[21] Liu, C., Kroeze, C., Hoekstra, A. Y., & Gerbens-Leenes, W. Past and future trends in grey water footprints of anthropogenic nitrogen and phosphorus inputs to major world rivers. *Ecological Indicators*, *18* (2012) 42–49. <https://doi.org/10.1016/j.ecolind.2011.10.005>.

[22] Canadian Council of Ministers of the Environment (CCME), Canadian Water Quality Guidelines for the Protection of Aquatic Life; CCME: Winnipeg, Canada, 2013.

[23] Allkem, *Balance hidrológico y modelo conceptual e hidrogeológico de superficie de la cuenca Olaroz-Cauchari.* Dirección de Minería y Recursos Energéticos de la provincia de Jujuy, Dirección de Recursos Hídricos de la provincia de Jujuy, Gerencias de Minera Exar S.A., Sales de Jujuy S.A. y South American Salars S.A., Jujuy, Argentina, 2022.

[24] Consejo Federal de Inversiones (CFI), *Estudio hidrogeológico Cuenca Río de los Patos – Salar del Hombre Muerto – Tomo I.* CONHIDRO S.R.L., Provincia de Catamarca, 2019.

[25] Consejo Federal de Inversiones (CFI), *Estudio hidrogeológico Cuenca Río de los Patos – Salar del Hombre Muerto – Segunda etapa. Informe Final.* CONHIDRO S.R.L., Provincia de Catamarca, 2021.

[26] Frau, D., Moran, B. J., Arengo, F., Marconi, P., Battauz, Y., Mora, C., Manzo, R., Mayora, G., & Boutt, D. F. Hydroclimatological patterns and limnological characteristics of unique wetland systems on the Argentine High Andean Plateau. *Hydrology*, *8*(4) (2021)164. <https://doi.org/10.3390/hydrology8040164>.

[27] World Health Organization & United Nations International Children's Emergency Fund (WHO & UNICEF), Progress on drinking water, sanitation and hygiene: 2017 update and SDG baselines. Geneva, Switzerland, 2017. [iris.who.int/bitstream/handle/10665/260291/9789243512891-spa.pdf?sequence=1&isAllowed=y](https://iris.who.int/bitstream/handle/10665/260291/9789243512891-spa.pdf?sequence=1&isAllowed=y).

1. Source: EIR file number IIA MT0655-134-2018 [↑](#footnote-ref-1)
2. Source: EIR file number M2650-2017 [↑](#footnote-ref-2)
3. Source: EIR file number IIA MT0655-134-2018 [↑](#footnote-ref-3)
4. Source: EIR file number M2650-2017 [↑](#footnote-ref-4)
